# Recovering Low-Density Polyethylene Waste for Gypsum Board Production: A Mechanical and Hygrothermal Study

**DOI:** 10.3390/ma17163898

**Published:** 2024-08-06

**Authors:** Alicia Zaragoza-Benzal, Daniel Ferrández, Paulo Santos, André Cunha, Luisa Durães

**Affiliations:** 1 Departamento de Tecnología de la Edificación, Universidad Politécnica de Madrid, Avda. Juan de Herrera, 6, 28040 Madrid, Spain; alicia.zaragoza@upm.es (A.Z.-B.); daniel.fvega@upm.es (D.F.); 2 University of Coimbra, Department of Civil Engineering, ISISE, ARISE, 3030-788 Coimbra, Portugal; andremac.cunha@gmail.com; 3 University of Coimbra, Department of Chemical Engineering, CERES, Rua Silvio Lima, 3030-790 Coimbra, Portugal; luisa@eq.uc.pt

**Keywords:** gypsum, low-density polyethylene (LDPE), hygrothermal performance, circular economy, sustainable construction

## Abstract

In recent decades, plastic waste management has become one of the main environmental challenges for today’s society. The excessive consumption of so-called single-use plastics causes continuous damage to ecosystems, and it is necessary to find alternatives to recycle these products. In this work, a mechanical and hygrothermal characterisation of novel plaster composites incorporating LDPE waste in their interior was carried out. Thus, prefabricated plasterboards have been designed with a partial replacement of the original raw material with recycled LDPE in percentages of 5–10–15% by volume. The results show how these new composites exceeded the 0.18 kN minimum breaking load in panels in all cases, while decreases in density and thermal conductivity of up to 15% and 21%, respectively, were obtained. In addition, an increase of 3.8%in thermal resistance was obtained by incorporating these new gypsum boards in lightweight façade walls through simulations. In this way, a new pathway was explored for the recovery of these wastes and their subsequent application in the construction sector.

## 1. Introduction

Global population growth and increasing urbanisation are forcing the implementation of new environmentally responsible economic growth strategies [1]. According to Agyeman et al., municipal solid waste will reach nearly 2.2 billion tonnes/year by 2025 [2]. In this sense, the construction industry is in a key position to move towards sustainable development and the design of new products made under circular economy criteria [3]. In recent decades, several researchers have worked on the development and characterisation of new construction products that incorporate secondary raw materials from urban waste [4,5]. Plastics have been one of the most widely used wastes in the manufacture of new construction materials. This is not surprising, as about 330 million metric tonnes of plastic waste is generated annually, of which only 9% is recycled [6]. Consequently, this plastic waste accumulates progressively, generating a strong environmental impact that represents one of the great challenges for today’s civilisation. It is therefore necessary to move towards new management models to reduce this waste, conserve natural resources, and regenerate and preserve the environment [7].

Plasterboards are widely used worldwide [8]. Their development has encouraged the implementation of modular construction systems that are less time consuming, economical, and environmentally friendly [9]. For this reason, in the last decade, several researchers have focused on the incorporation of plastic waste as an addition in the manufacturing process of these gypsum composite materials. In Table 1, a review of the current existing literature is displayed, schematically showing the studies that have been carried out.

As can be seen in Table 1, different studies with plastic wastes integrated in gypsum composites have been conducted in the last decade. Firstly, due to the low thermal conductivity of plastic materials, several authors [15,16,18,21] have tried to improve the thermal resistance of traditional gypsum materials. Thus, the incorporation of these wastes as a partial replacement of the original gypsum material allows for reducing the thermal conductivity of the prefabricated gypsum [16,17], contributing to improve the energy efficiency of the construction systems where prefabricated boards are integrated. On the other hand, as a consequence of the impermeable properties of plastics, other research [11,18,25] has focused on improving the water resistance of gypsum composite materials. Thus, replacing the original raw material with recycled plastic aggregates reduces capillary water absorption and total water absorption [18,25]. This effect has been shown to have a positive impact on increasing the durability of prefabricated gypsum housing products [11,14], making it one of the main applications of these secondary raw materials in building construction.

Regarding the mechanical properties of composites included in Table 1, a decrease in strength is observed in comparison with traditional gypsum materials. Thus, related to the decrease in density of hardened gypsum, the incorporation of plastic residues reduces mechanical compressive strength [11,21,22,25,26]. Nevertheless, in all cases included in Table 1, 2 MPa strength was exceeded. In terms of flexural strength, there are different results depending on the morphology of the plastic waste added. Thus, in those investigations where the residue was incorporated in fibre form, the flexural strength increased with respect to the reference material [12,22,23], which did not occur when additions in aggregate form were used [18,20,21,25]. This is due to the fact that these plastic aggregates generate preferential breaking points in the gypsum composite matrix under bending stresses. However, in the clear majority of studies [15,18,19], there is a good integration between the plastic aggregate and the matrix, which is evidenced by the formation of dihydrate at the matrix–plastic aggregate interface. Thus, it can be stated that all materials included in Table 1 were found to be suitable for building applications, and those that were tested for their feasibility in the development of prefabricates [14,16,17] showed their suitability for moving towards a circular construction industry.

For the specific case of this research, the study of gypsum composite materials with partial substitution of the original raw material by shredded LDPE waste from single-use bags is addressed. The accumulation of LDPE waste poses a threat to the world’s terrestrial, marine, and freshwater ecosystems [27]. According to recent research, most plastics disposed of worldwide are composed of LDPE [28]. The non-biodegradable nature of this LDPE waste exacerbates the problem arising from the current inefficient management of these materials and contributes to a persistent environmental problem [29]. Currently, about 100 billion polyethylene bags are used in different sectors, approximately 177 units/person, and only 7% are recycled [20]. Thus, it is one of the most inefficient products in the world, with an average usage time of 15 min and a decomposition time in nature of more than 50 years [30]. Therefore, any effort to integrate these wastes into the development of new circular construction products contributes to moving towards a sustainable and environmentally committed construction sector [29].

The original idea to conduct this research work arose from the following widely known premise: gypsum composites have a good capacity for hygrothermal regulation [31], as well as a flexural and compressive strength that decreases as the mixing water content increases [32]. In this sense, the question emerges as to how the incorporation of these LDPE plastic wastes affects the hygrothermal and mechanical behaviour of gypsum-based materials. Thus, the aim of this research is to analyse the hygrothermal and mechanical properties of a novel prototype gypsum-based material designed for application in the manufacture of prefabricated plasterboards. This would allow for the development of these novel construction materials incorporating LDPE waste in the composite matrix to be promoted as a possible solution to the recycling and revalorisation of single-use plastic products. For this purpose, a detailed experimental campaign was carried out in different phases and, finally, a critical discussion of the application possibilities of these sustainable building materials is presented. Therefore, this research will provide a new approach to producing prefabricated construction plates and panels, showing the benefits derived from the incorporation of this plastic waste in terms of improved thermal behaviour, lightening, and the study of the properties of gypsum in relation to the action of water.

## 2. Materials and Methods

In this section, the methodology followed to conduct this research is described. This includes a description of the materials, sample preparation, and experimental programme developed.

### 2.1. Employed Materials

The following raw materials were used to produce the gypsum composites: building gypsum, drinking water, LDPE waste, and Kraft paper.

The binder used was a construction gypsum of type YF-B1, according to the designation of the UNE-EN 13279-1 standard [33]. This material is commonly used to produce prefabricated and finished housing, and has been successfully used in other research work [17,23,26]. The main characteristics of this material are listed in Table 2. This information has been provided by the manufacturer Saint-Gobain Placo Ibérica S.A. (Madrid, Spain).

For mixing of the gypsum composites developed in this research, drinking water from the Canal de Isabel II (Community of Madrid, Spain) was used, in accordance with European Council Directive 98/83/EC [34]. This water is characterised by a pH between 7.5 and 8.0, average hardness of 25 mg CaCO_3_ per litre, and chlorine content between 1.0 and 1.5 mg/L [35].

**Figure 1:**
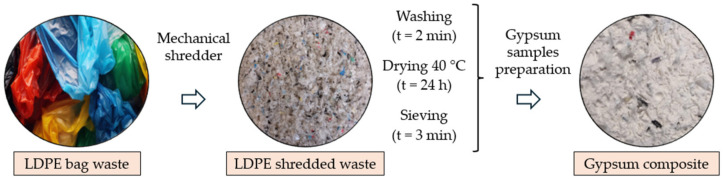
Preparation process of LDPE waste for use in gypsum composites.

The recycled material used as partial replacement for the original gypsum compound was shredded low-density polyethylene (LDPE). This material was extracted after a single-use bag shredding process, as shown in Figure 1.

After shredding, washing, drying, and sieving of LDPE waste, particle sizes between 0.125 and 1.000 mm were obtained, according to standardised sieve series of UNE-EN 933-2 standard [36]. A mechanical plastics shredder equipped with steel blades was used to obtain the scraps used. The most relevant properties of these recycled materials are [20] a melting temperature of 105–115 °C, tensile strength of 30 MPa, elongation to fracture of 400%, thermal conductivity of 0.5 W/m∙K, and real density of 930 kg/m^3^.

Finally, to reinforce the gypsum composite materials produced, a series of plates were produced for flexural testing, reinforced with Kraft paper. These paper coatings provide gypsum prefabricated products with greater rigidity and mechanical strength [37]. This material has a density of 45 g/m^2^, brown colour, and is fully recyclable.

### 2.2. Sample Preparation

**Figure 2:**
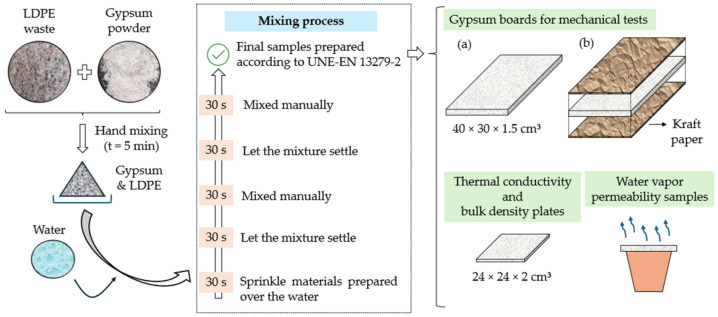
Simplified outline of the sample preparation process: (**a**) Gypsum board without Kraft paper; (**b**) Gypsum board with Kraft paper.

The mass proportions used for the preparation of the composites developed in this work are shown in Table 3. It should be noted that for the mixing process of samples, the recommendations of the UNE-EN 13279-2 standard [38] were followed, the process of which is shown in Figure 2. It should be noted that this mixing process was carried out manually, following the indications of the mentioned standard.

Regarding the dosages shown in Table 3, a saving in natural resource consumption can be seen when replacing the original gypsum raw material with LDPE waste. Thus, for the specific case of this research, substitution percentages of up to 15% by mass were used. This percentage has been considered the limit to obtain a good workability of the mixture with the water/gypsum ratio 0.7 by the mass selected. All the composites produced showed a diameter of 165 ± 10 mm after the shaking table test. On the other hand, a decrease in the setting time of the mixes, determined according to the Vicat needle method, can be observed. Specifically, for the mixture with the highest plastic residue content, the setting time was reduced by 15% compared to the reference material (G0.7). This effect can be a competitive advantage to increasing factory productivity during the manufacture of precast plates and panels [39].

On the other hand, the elaboration process shown in Figure 2 was designed in accordance with the recommendations and times included in the UNE-EN 13279-2 standard [38]. The samples were cured for seven days, after which they were subjected to a 24 h drying process prior to testing at a temperature of 40 ± 2 °C and a relative humidity of 50 ± 5%.

### 2.3. Experimental Programme

The tests carried out to develop this experimental programme, as well as the reference standards, are described below:

- **Flexural strength**: determined according to indications of the UNE-EN 12859 standard [40]. It consists of determining the maximum breaking load that plasterboards subjected to a three-point pure bending test are able to withstand. Plates with dimensions of 40 × 30 × 1.5 cm^3^ are placed 35 ± 1 cm apart on the rollers of the PÁCAM model MPX-22 press, after which a load is applied in the centre of the span until the piece breaks. A total of three specimens were tested for each type of gypsum material studied. It should be noted that the plates were sanded on their surface to obtain a constant average thickness of 1.5 cm for all composites.
- **Surface hardness**: determined with the aid of a Shore C durometer according to UNE 102042:2023 [41] recommendations. This property reflects the material’s resistance to scratching on its surface. For this purpose, a total of ten measurements were taken per plate, with each measurement spaced at least two centimetres apart and between the measurement and the edges of the plate.
- **Scanning electron microscopy (SEM)**: This test was carried out to determine the microstructural behaviour of the analysed composites. For this purpose, a Jeol JSM-820 microscope (Jeol, Croissy-sur-Seine, France) operating at 20 kV, equipped with Oxford EDX analysis, was used. The fragments analysed were extracted from the interior of the samples without modifying their surface texture. Using a Cressington 108 metalliser (Cressington, Watford, UK), the test samples were coated with a thin layer of gold foil to ensure good conductivity to the electron beam generated by the equipment.
- **Bulk density**: physical property determined according to UNE 102042:2023 [41], using 24 × 24 × 2 cm^3^ plates and a 0.01 g precision balance. A total of three samples of each gypsum type included in this study were tested.
- **Thermal conductivity**: obtained using a mini Hot-Box (DEC-FCTUC, Coimbra, Portugal), equipped with thermocouples connected to a computer to record the data [32]. The test was carried out using 24 × 24 × 2 cm^3^ gypsum plates, placed on one side of the Hot-Box in such a way that the heat flow migrated from the inside to the outside; the thermal conductivity was measured 24 h once the steady state was reached, and, finally, the Fourier equation was applied (1):
  (1) $$\Phi =\frac{\lambda }{e}\cdot S\cdot \left(T_{int}-T_{ext}\right)$$
  where Φ represents the heat flow; λ is the coefficient of thermal conductivity of the material under test; e represents the plate thickness and S its surface; and $T_{int}$ y $T_{ext}$ are the inside and outside temperature of the mini Hot-Box, respectively. A total of three samples were analysed for each type of gypsum.
- **Water vapour permeability**: determined according to the recommendations of the UNE-EN ISO 12572 standard [42]. For this purpose, circular samples with a diameter of 10 cm and a thickness of 1.0 ± 0.1 cm were used. These samples were placed covering a recipient containing a saline solution inside and sealed with silicone. As mentioned above, the recipient contained a saturated solution of potassium nitrate (KNO_3_) in water. Thus, for a period of eight weeks, the samples were weighed weekly in order to relate the mass variation to the water passing through the gypsum compound under study in the form of vapour. Thus, the water vapour permeability was determined by the expression (2):
  (2) P=PR·e
  where P is the water vapour permeability, e is the thickness of the plaster sample, and PR is the water vapour permeance. *PR* is determined by Equation (3):
  (3) $$PR=\frac{WVT}{∆p}$$
  where WVT represents the water vapour transmission rate determined by Equation (4) and ∆p is a pressure-dependent coefficient given by Equation (5).
  (4) $$WVT=\frac{∆m}{t\cdot A}\;$$
  (5) $$∆p=S\cdot \left(R_{1}-R_{2}\right)$$
  ∆m is the mass variation, t is the time between measurements, A is the sample area, S is the saturation pressure of water vapour at the test temperature (18.663 mmHg at 21 °C), $R_{1}$ is the relative humidity on the side of the sample with the highest vapour pressure (94% inside the container), and $R_{2}$ is the relative humidity in the environment (50% in laboratory conditions).

Finally, a critical review and potential application of these novel gypsum composite materials is included. For this purpose, a comparative analysis was carried out based on the properties analysed in this research, as well as a simulation of the thermal performance of these composite materials for their application in buildings. The latter was conducted using the THERM simulation software (version 7.8), a two-dimensional heat transfer computational tool widely used in the building sector [43].

## 3. Results and Discussion

This section presents the results obtained for the mechanical and hygrothermal characterisation of the gypsum composites developed in this research. Furthermore, the results are discussed based on other research works, and the potential application of these novel composite materials is explored.

### 3.1. Mechanical Tests on Prefabricated Plates

**Figure 3:**
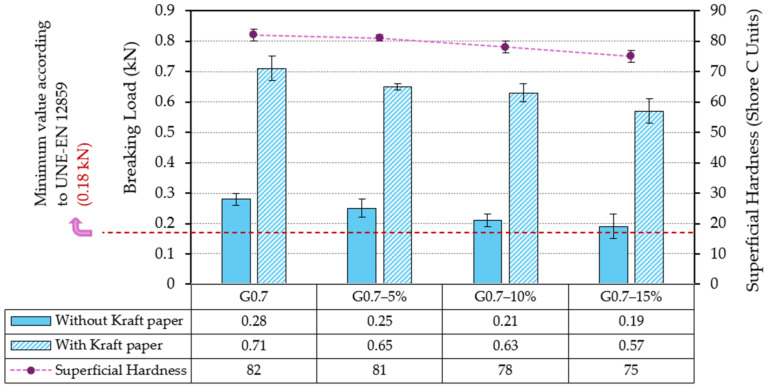
Mechanical test results on gypsum plates (UNE-EN 12859 [40]).

Figure 3 shows the results obtained for the flexural strength test on plates and for the surface hardness of the gypsum composites prepared.

As can be seen in Figure 3, the mechanical properties are progressively reduced as the recycled LDPE content in composites increases. Thus, the surface hardness of G0.7-15% composite decreases by 8.5% with respect to traditional gypsum composite. According to several authors [11,21], this decrease in hardness caused by the partial replacement of gypsum by LDPE waste is linked to a lower density of the material and a reduction in compressive strength.

On the other hand, in the flexural strength tests carried out on prefabricated plates, all composites analysed exceeded the minimum breaking load of 0.18 kN established by current regulations. However, strong differences are observed between series with and without Kraft paper reinforcement. Thus, for the composite with the highest content of recycled material (G0.7-15%) and without reinforcement, the maximum breaking load reached was 0.19 kN. However, with the surface bonding of Kraft paper sheets, the maximum load supported increases to 0.54 kN (more than three times higher than the minimum recommended value). These results suggest the feasibility of applying these materials to the design of building prefabricated units, with values in agreement with those obtained in other studies, as shown in Table 4.

**Figure 4:**
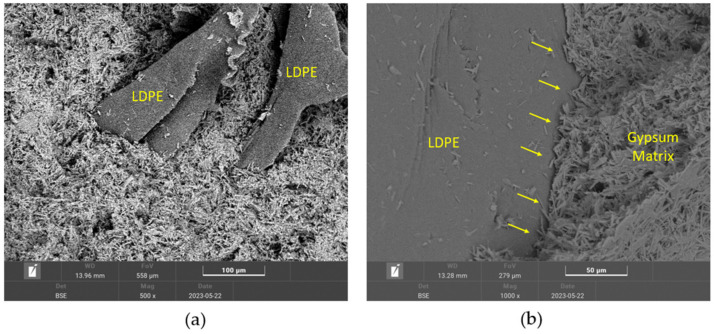
SEM analysis for sample G0.7-15%, magnifications: (**a**) ×500; (**b**) ×1000.

Additionally, to complement the mechanical characterisation, a SEM analysis of the sample with the highest plastic residue content was performed. Figure 4 shows the microstructure of the composite G0.7-15%.

Figure 4a shows the characteristic acicular morphology of dihydrate crystals (CaSO_4_·2H_2_O), which is indicative of a correct setting process of the gypsum material [32]. Thus, the added LDPE residues do not condition the normal hardening process of the sample. It can be observed that these plastic residues are perfectly integrated in the matrix of the composite. This can be seen in more detail in Figure 4b, where the gypsum–LDPE interface is shown, with good adhesion between the two materials. This integration is essential to avoid residue slippage and subsequent detachment after breakage [48].

### 3.2. Thermal Behaviour of Elaborated Gypsum Composites

**Figure 5:**
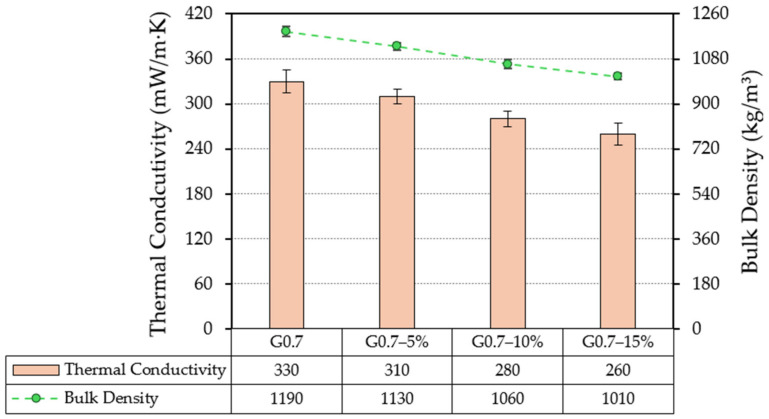
Results for thermal conductivity and bulk density of gypsum composites.

In this section, the impact of replacing the original gypsum raw material with LDPE waste on the thermal conductivity and bulk density of the composites produced is discussed. The results obtained are shown in Figure 5.

Firstly, Figure 5 shows how reducing gypsum content and replacing it with shredded LDPE waste results in a decrease in the bulk density of composites. Thus, in the case of sample G0.7-15%, the decrease in density was 15% with respect to the reference material (G0.7). Therefore, as highlighted in other research works, the partial substitution of the original raw material by lightweight plastic waste can be a potential source of competitive advantage for construction companies: (i) by reducing transport costs [14], and (ii) by reducing execution and placement times of on-site prefabricated systems [16].

Similarly, in Figure 5, a decrease in thermal conductivity is observed as the LDPE waste content in the gypsum samples increases. Related to its lower density and thermal conductivity coefficient, the LDPE waste used allowed the G0.7-15% sample to have a thermal conductivity 21% lower than the reference (G0.7) [49]. These results suggest the potential application of these novel building materials to improve the energy efficiency of construction systems where they are applied. Several authors agree that this is perhaps the main advantage of incorporating this type of plastic addition in gypsum composites [50]. In addition, it is corroborated that results obtained in this research are consistent with those obtained in other studies, as shown in Table 5.

### 3.3. Water Vapour Permeability

**Figure 6:**
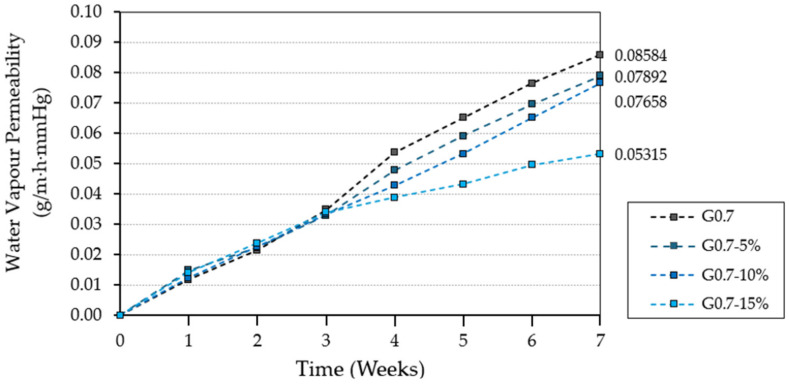
Water vapour permeability of gypsum composites analysed.

Water vapour permeability is a property of particular interest for gypsum composites, which are subject to constant changes in ambient humidity. The results obtained for this analysis are shown in Figure 6.

Water vapour permeability in porous materials is affected by the moisture present in the material and the ambient humidity [51]. For this reason, all tested samples were previously dried (24 h at 40 ± 2 °C) and tested in laboratory conditions with an ambient relative humidity of 50 ± 5%. As can be seen in Figure 6, the incorporation of LDPE waste in gypsum composites decreases their ability to diffuse water vapour through them. Thus, the sample with the highest content of recycled material G0.7-15% reduced its permeability to water vapour by 37.6% with respect to the reference material G0.7. Therefore, these new composites would have a potential application in humid rooms, where humidity damping is essential to avoid the development of pathologies in dwelling interiors [52].

### 3.4. Critical Discussion and Future Applications

The construction sector generates about 36% of total solid waste and consumes about 40% of the energy produced in the EU [53]. In view of this alarming situation, it is necessary to search for alternative solutions that are more energy efficient and developed under circular economy criteria. Thus, this work contributes to the redesign of traditional manufacturing processes of prefabricated products for lightweight partition walls and ceiling panels.

In general terms, the existing prefabricated plates and panels on the market are lightweight, made with synthetic reinforcing fibres and with different finishes depending on the environment in which they will be exposed. However, these commercial lightened gypsum products employ chemical additives to some extent to accelerate the setting processes, while they are manufactured with natural raw materials and rarely incorporate recycled products that do not come from waste generated in the factory itself [9].

**Figure 7:**
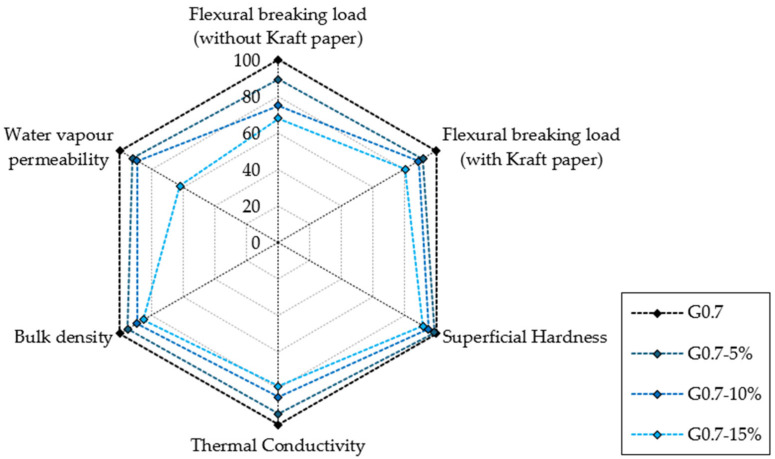
Comparative analysis of the different compounds.

In this sense, this research contributes to enhancing the use of secondary raw materials in the development of new sustainable construction products. Thus, through the recovery and revaluation of solid urban waste, such as shreds of LDPE from single-use bags, it has been possible to design novel gypsum composites that are suitable for precast production and more energy efficient than traditional gypsum composites. Figure 7 shows the comparative percentage variation in each of the analysed properties, so that it is possible to see the effect that the incorporation of the different percentages of LDPE causes with respect to the reference material (G0.7).

As shown in Figure 7, in no case was the maximum flexural ultimate load presented by the composite without additions exceeded. However, since these are non-structural elements and the minimum strength requirement of the current regulations has been guaranteed, the composites developed would be suitable for use in buildings. Especially in damp rooms, where their greater resistance to the passage of water vapour could represent a comparative advantage with respect to other products currently on the market.

**Figure 8:**
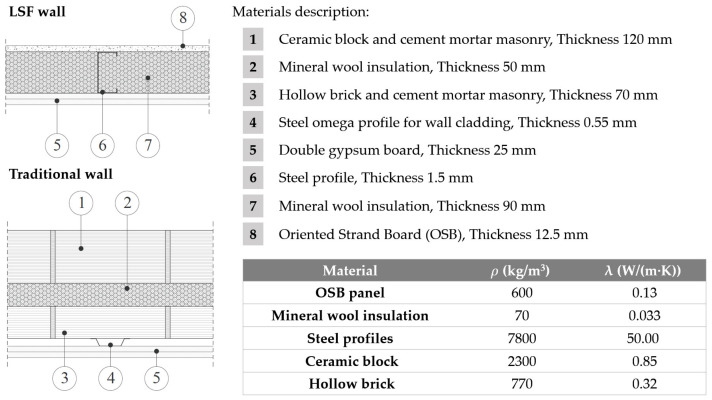
Description of the two construction systems used in the simulation and physical properties of the incorporated materials (excluding gypsum boards already shown in Figure 5).

**Figure 9:**
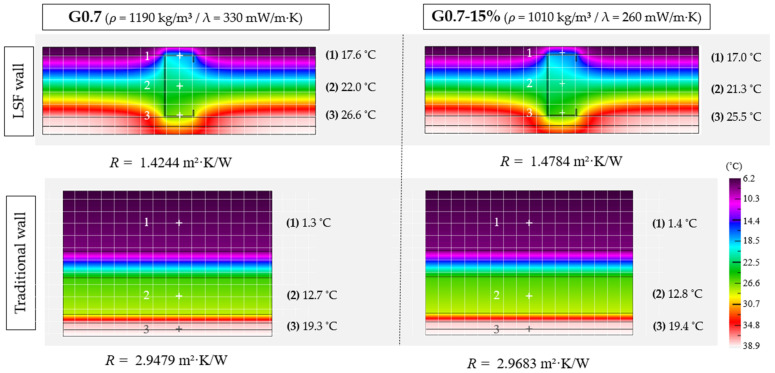
THERM simulation of two building systems (LSF wall and traditional facades) with interior plaster wall finish.

However, the greatest benefit of incorporating LDPE waste in gypsum composites manufacturing is the decrease in bulk density and the thermal conductivity of the hardened material. These obtained results correspond to those expected in the design of this research, since in general terms, the incorporation of recycled plastic materials weakens the matrix of gypsum composites, reducing their mechanical strength [54]. However, these recycled materials also make it possible to obtain more impermeable and lighter composites, which is expected to increase the overall thermal resistance of these composite materials compared to traditional ones [55]. A simulation performed with THERM software (version 7.8) on two construction systems (conventional facade and Lightweight Steel Frame (LSF) wall), as described in Figure 8, revealed the effect of this improvement in thermal properties, as shown in Figure 9.

As can be seen in Figure 9, for both types of construction systems there is a slight improvement in the final thermal resistance of the building envelope. In the LSF wall, by incorporating the G0.7-15% plaster sheets, the thermal resistance of the wall increases by 3.8% with respect to the reference, with this difference not being so significant in the case of the traditional wall. The temperatures inside the wall with the G0.7-15% plaster are lower than in the reference wall, since the lower thermal conductivity of the new compound insulates the inside of the wall more efficiently. This results in a lower amount of energy dissipated through the wall, increasing the energy efficiency of the system. No significant changes were observed in the traditional wall, since the air layer between the wall and the G0.7-15% panels reduces the effect of the new material on the whole. Thus, the influence of the plates based on the new composite becomes more relevant in lightweight systems with reduced thicknesses, where the thermal conductivity of each layer contributes to the energy performance of the facade to a greater extent.

In any case, not only does it contribute to improving the energy efficiency of buildings, but also, thanks to the reduction in the final density of the composites, lighter, more economical, and technically feasible prefabricated products are obtained. This decrease in density, which makes the execution of lightened slabs and panels possible, is in turn beneficial for the operator, who enjoys a reduced effort required for their placement in situ, increasing their productivity and reducing the risk of injuries due to the mobility of heavy parts [56]. In the same way, this effect on the final lightening of the precast elements would lead to savings in transportation costs, as well as a decrease in CO_2_ emissions imputed to the logistics process, which is environmentally beneficial. However, there is still a long way to go to be able to commercialise this type of product, which, although it is true that it can deliver a competitive advantage in costs compared to those traditionally used, still needs a process of commercial establishment and a progressive mindset change in consumers, which will be supported by the impulse of new European regulations [57]. 

## 4. Conclusions

This research has explored a novel method for the recovery and revalorization of LDPE waste from single-use bags. Thus, an experimental campaign has been carried out to analyse the mechanical and hygrothermal properties of some novel prefabricated plaster with the incorporation of LDPE waste. The most relevant conclusions that can be drawn from this research, where a saving of up to 15% of the original raw material has been achieved, are as follows:

- No excessive decrease in surface hardness is observed when incorporating LDPE waste in gypsum composites. The maximum reduction is 8.5% for G0.7-15% composite.
- The maximum flexural ultimate load is progressively reduced as the original plaster material is replaced by recycled LDPE. This is due to the weakening of the original composite matrix as it is replaced by the waste. For the samples without Kraft paper, the strength was reduced by up to 32%. However, with the adhesion of Kraft paper, the strength of the G0.7-15% composite resisted up to a maximum load of 0.57 kN, which is three times higher than the minimum value set by the standard at 0.18 kN.
- SEM analysis evidenced a perfect integration and distribution of the LDPE residue in the gypsum matrix.
- The replacement of the original gypsum material by LDPE residue allows for a reduction in the final density of the composites in hardened state.
- The thermal conductivity of the reference material (G0.7) was reduced by 21% for the sample with the highest recycled plastic content.
- Water vapour permeability was reduced because of the incorporation of LDPE waste in the gypsum matrix. This decrease was up to 37.5% for the G0.7-15% composite compared to the G0.7 sample of traditional mortar.
- The simulations performed with THERM tool show the goodness of gypsum composites with LDPE waste incorporation to increase the thermal resistance in LSF wall facades.

Finally, it should be noted that for a complete characterisation of these prefabricated products, it would be convenient to carry out studies on the acoustic behaviour, as well as on the fire behaviour, of the gypsum materials manufactured. These tests would provide useful information to manufacturers for the possible commercialization of these products. It would also be interesting to carry out durability studies to analyse the compatibility between the two materials and their different decomposition processes over time, which would guarantee that the use of these prefabricated products meets the recommended quality and durability standards. Likewise, a life cycle analysis would make it possible to quantify the potential improvement in terms of CO_2_ equivalent emissions of reducing the consumption of the binder in the production phase.

## Figures and Tables

**Table 1:** Literature review: gypsum composites with added plastic waste *.

Ref.	Binder	Waste **	Size	Addition	Experiments ***																	
					A	B	C	D	E	F	G	H	I	J	K	L	M	N	O	P	Q	R
[10]	Plaster	Cable plastic	<3 mm	50–60–70 wt.%	•	•	•	•														
[11]	Plaster	Cable plastic	<3 mm	50–60–70 wt.%.		•			•	•	•	•	•	•	•							
[12]	Gypsum	Nylon fibre	20–25 mm	2.5 wt.%					•	•	•					•	•	•				
		PP	<4 mm	7.5 wt.%					•	•	•					•	•	•				
[13]	Gypsum	Nylon fibre	20–25 mm	2.5 wt.%			•	•								•	•		•	•	•	
		PP	<4 mm	7.5 wt.%			•	•								•	•		•	•	•	
[14]	Gypsum	Nylon fibre	20–25 mm	2.5 wt.%	•				•	•	•			•		•						•
		PP	<4 mm	7.5 wt.%	•				•	•	•			•		•						•
[15]	Gypsum	Polycarbonate	<4 mm	10–20–30–40 wt.%.	•		•			•	•					•						
[16]	Plaster	Cable plastic	<3 mm	50–60–70 wt.%	•				•	•							•					•
[17]	Gypsum	CDs and DVDs	0.25–4 mm	17–35–70 vol.%	•			•		•						•						•
[18]	Plaster	HDPE	1.4	2–4–6–8–10 vol.%	•			•	•	•	•			•	•	•	•					
[19]	Gypsum	PP	0.13–4 mm	2.5–5–7.5–10 wt.%				•	•	•	•	•	•		•	•	•					
[20]	Plaster	LDPE	0.125–1 mm	1–2–3 wt.%	•			•	•	•	•			•	•	•						
[21]	Plaster	EPS	1–4 mm	0.01–0.05–0.1 wt.%	•				•	•	•					•						
[22]	Gypsum	PEFN fibre	20–30 mm	0.25–2.0 wt.%				•		•	•								•			
[23]	Plaster	Polyester fibre	30 mm	1 wt.%					•	•	•				•	•						
[24]	Plaster	EPS and XPS	1–4 mm	1–2–3–4 wt.%					•	•	•					•						
[25]	Plaster	ELT rubber	<4 mm	30–40–50–60 wt.%.			•	•						•	•							
[26]	Gypsum	Polyurethane	<0.5 mm	50–100–200 vol.%.	•			•	•	•				•		•		•				

* Search in Web of Science: ((“Gypsum*” OR “Plaster*” OR “Plasterboard*”) AND (“Plastic*” OR “Plastic Waste”)). ** Waste: Polypropylene (PP), High-Density Polyethylene (HDPE); Expanded Polystyrene (EPS); Polyethylene Fishing Net (PEFN); Extruded polystyrene (XPS); End-of-Life Tyre (ELT). *** Experiments: (A) Thermal conductivity; (B) Moisture content; (C) Porosity; (D) Scanning Electron Microscopy; (E) Superficial hardness; (F) Flexural strength; (G) Compressive strength; (H) Water vapour permeability; (I) Water–Stove cycles; (J) Water absorption; (K) Capillarity test; (L) Bulk density; (M) Young Modulus; (N) Fire reaction; (O) X-ray Diffraction; (P) Thermogravimetric Analysis; (Q) Higher temperature test; (R) Precast validation. The black dots “•” mean accomplished experiments.

**Table 2:** Technical characteristics of the construction plaster YF-B1.

Water Vapour Diffusion Factor	Flexural Strength (MPa)	Compressive Strength (MPa)	pH
6	≥1	≥2	>6
**Fire reaction (Euroclass)**	**Granulometry (mm)**	**Purity index (%)**	**Usage time (min)**
A1	0–0.2	80	15–20

**Table 3:** Dosages used to produce the gypsum compounds studied (mass ratios).

Type	Gypsum (g)	Water (g)	LDPE (g)	Raw Material Savings (%)	Setting Time (min)
G0.7	1000	700	—	—	22
G0.7-5%	950	665	20	5	21
G0.7-10%	900	630	40	10	18
G0.7-15%	850	595	60	15	17

**Table 4:** Literature review: values obtained for flexural breaking load and surface hardness in other studies ^(^*^)^.

Property	[44]	[45]	[17]	[16]	[18]	[46]	[47]
Flexural breaking load (without Kraft paper) [kN]	0.16	0.34	0.47	0.18	0.16	0.52	0.25
Superficial hardness (Shore C Units)	73	88	—	78	73	63	89

^(^*^)^ Dosages: G0.65 with 30% volume substitution of ELT recycled rubber [44]; G0.7 with 2% weight substitution by ELT textile fibres [45]; G0.65 with 71% volume substitution by CDs [17]; G0.8 with 80% volume substitution by plastic cable waste [16]; G0.65 with 10% volume substitution by HDPE aggregates [18]; G0.7 with 28% mass substitution by EPS solution [46]; and G0.7 with 1.5% mass addition of potassium polyacrylate [47].

**Table 5:** Values obtained for thermal conductivity and bulk density in other research ^(^*^)^.

Property	[10]	[15]	[18]	[21]	[26]	[44]	[46]
Thermal conductivity (W/m·K)	0.25	0.18	0.19	0.12	0.25	0.17	0.11
Bulk density (kg/m^3^)	1023	1019	1040	610	753	1060	833

^(^*^)^ Dosages: G0.8 with 80% volume substitution by plastic cable waste [10]; G0.55 with 40% volume substitution by polycarbonate waste [15]; G0.65 with 10% volume substitution by HDPE recycled aggregates [18]; G0.8 with 0.1% addition by weight of recycled EPS [21]; G0.6 with 100% addition by volume of polyurethane waste [26]; G0.65 with 30% volume substitution by ELT rubber aggregates [44]; and G0.7 with 28% mass substitution by EPS solution [46].

## Data Availability

All data have been included in the manuscript.
